# Is Active Management the Key to the Conservation of Saproxylic Biodiversity? Pollarding Promotes the Formation of Tree Hollows

**DOI:** 10.1371/journal.pone.0060456

**Published:** 2013-03-27

**Authors:** Pavel Sebek, Jan Altman, Michal Platek, Lukas Cizek

**Affiliations:** 1 Institute of Entomology, Biology Centre of the Academy of Sciences of the Czech Republic, Ceske Budejovice, Czech Republic; 2 Faculty of Science, University of South Bohemia, Ceske Budejovice, Czech Republic; 3 Institute of Botany of the Academy of Sciences of the Czech Republic, Pruhonice, Czech Republic; Roehampton university, United Kingdom

## Abstract

Trees with hollows are key features sustaining biodiversity in wooded landscapes. They host rich assemblages of often highly specialised organisms. Hollow trees, however, have become rare and localised in Europe. Many of the associated biota is thus declining or endangered. The challenge of its conservation, therefore, is to safeguard the presence of hollow trees in sufficient numbers. Populations of numerous species associated with tree hollows and dead wood are often found in habitats that were formed by formerly common traditional silvicultural practices such as coppicing, pollarding or pasture. Although it has been occasionally mentioned that such practices increase the formation of hollows and the availability of often sun-exposed dead wood, their effect has never been quantified. Our study examined the hollow incidence in pollard and non-pollard (unmanaged) willows and the effect of pollarding on incremental growth rate by tree ring analysis. The probability of hollow occurrence was substantially higher in pollard than in non-pollard trees. Young pollards, especially, form hollows much more often than non-pollards; for instance, in trees of 50 cm DBH, the probability of hollow ocurrence was ∼0.75 in pollards, but only ∼0.3 in non-pollards. No difference in growth rate was found. Pollarding thus leads to the rapid formation of tree hollows, a habitat usually associated with old trees. It is therefore potentially a very important tool in the restoration of saproxylic habitats and conservation of hollow-dependent fauna. If applied along e.g. roads and watercourses, pollarding could also be used to increase landscape connectivity for saproxylic organisms. In reserves where pollarding was formerly practiced, its restoration would be necessary to prevent loss of saproxylic biodiversity. Our results point to the importance of active management measures for maintaining availability, and spatial and temporal continuity of deadwood microhabitats.

## Introduction

In Europe, the intensification of forestry and agriculture and the abandonment of traditional silvicultural practices have resulted in an increase in the size of landscape mosaic grain, increased canopy closure, and a decline in the number of old and open-grown trees in both forested and agricultural landscapes [1]–[4]. Old trees with hollows (i.e. cavities) are a key feature in sustaining biodiversity in wooded landscapes because they host rich, specialised assemblages of numerous vertebrate and invertebrate species [5]–[7]. Their walls and wood mould (loose decayed wood that accumulates in hollows) provide a continuous supply of dead wood to wide spectrum of saproxylic (dead wood dependent) invertebrates and fungi that constitute a large part of woodland biodiversity [8], [9]. Some, such as the hermit beetle *Osmoderma eremita*, are found only in tree hollows, while others exploit other deadwood microhabitats such as old and dead trees, including the stag beetle *Lucanus cervus*, and the Rosalia longicorn *Rosalia alpina* [10], [11].

These key deadwood habitats, such as old trees and hollow trees, are now rare and localised in Europe and most organisms associated with them are thus threatened or declining. Their conservation requires that the continuity of these deadwood habitats is maintained [3], [12]–[14]. Knowledge about the formation of hollows is, therefore, needed. Several works have studied or simulated the occurrence of hollow-bearing trees at the stand or landscape level [15]–[18]. Because the natural formation of hollows takes a long time, active measures are often required to bridge the gap between current hollow trees and those young trees that will substitute for them in the future. Jansson *et al.* (2009) [19] suggest using special boxes that mimic tree hollows to help preserve endangered populations of hollow-dependent beetles. Bird nest boxes are commonly used to help hollow-nesting birds overcome the same problem. Such artificial hollows, however, are only able to support a small proportion of tree hollow organisms and their effect is relatively short-term. A simple method designed to speed up the formation of hollows is therefore needed.

Pollarding, i.e. the periodical removal of the upper branches of a tree by pruning, is an age-old practice [20]. It allows for the harvesting of firewood or fodder from trees without killing them. The branches are usually removed at a height that prevents herbivorous mammals from damaging resprouting shoots, thus allowing pasture and wood production to continue in conjunction. Stands of pollarded trees usually host rich assemblages of saproxylic species, many of which prefer sunny conditions, including endangered hollow specialists (e.g. *Osmoderma eremita*) [21], and other saproxylics (e.g. *Rosalia alpina*) [22], [23]. Some authors have mentioned the potential of pollarding for conservation, as pollard trees offer exposed wood and form tree hollows at a smaller diameter than non-pruned trees of the same species [24]–[27], and pollarding has already found its way into conservation practice [6], [22], [24], [27]. However, the effects of pollarding on the formation of hollows have not been studied yet.

If pollarding substantially increases the probability of hollow formation, it could serve as a key management tool for conserving the often highly endangered biota associated with them. In the present study, the hypothesis that pollarding affects the formation of tree hollows was tested by comparing hollow occurrence in pollards and in unmanaged (unpollarded) trees. To investigate the effects of pollarding on growth rate, patterns in annual ring increments were compared between pollards and unmanaged trees. To quantify the potential effects of pollarding on tree hollow availability, the probability of hollow occurrence in relation to tree diameter was predicted for pollards and unmanaged trees.

## Materials and Methods

### Study sites and data collection

**Figure 1:**
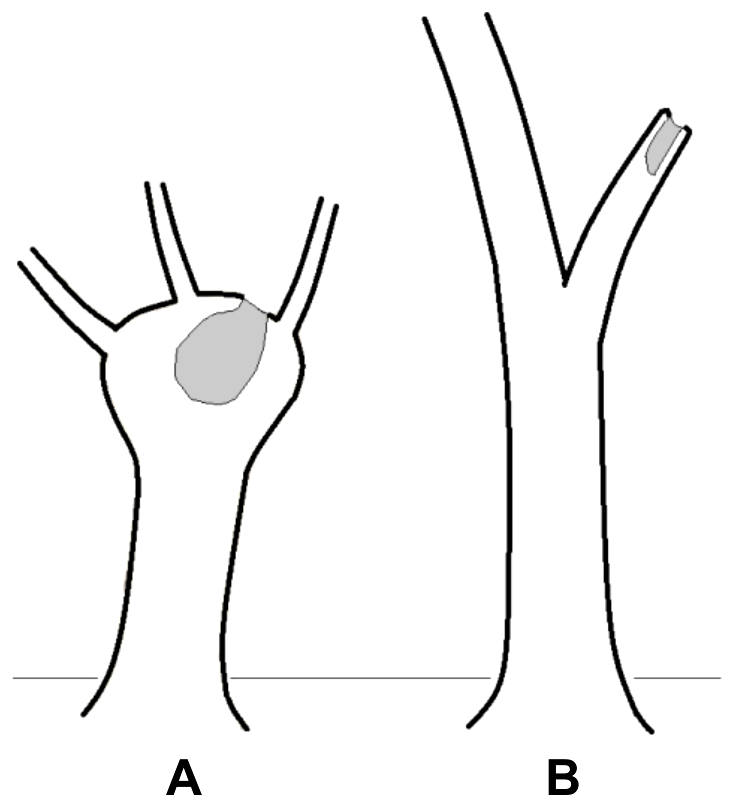
Forms of pollard and unmanaged trees. A pollard (A) and an unmanaged tree (B), shown with their most common type of tree hollows (grey colored): hollows formed in the upper parts of the trunk as a result of bared heartwood after pruning are common in pollards, whereas hollows formed after a branch fall are the most common hollow type in unmanaged trees.

Data were collected in stands of pollard and unmanaged (i.e. unpollarded) white willows (*Salix alba*) in the catchment area of the Thaya (Dyje) river in South Moravia, Czech Republic. Two stands of pollard trees, Vojkovice (49°3′N, 16°36′E) and Krive jezero (48°51′N, 16°43′E), and two stands of unmanaged willow trees, Kanci obora (48°46′N, 16°52′E) and Pastvisko (48°48′N, 16°47′E), were sampled. Vojkovice and Kanci obora are located on publicly accessible land, two other sites are located within protected areas. Site selection was constrained by the fact that pollarding of willows had been regionally common in the past, but was mostly discontinued after the Second World War. Old willows thus bear the signs of previous pollarding, whereas younger trees were (almost) never pollarded throughout the area. The areas where young trees were pollarded, or where older trees were not pollarded were therefore carefully selected. Trees with trunks higher than 0.5 m, whose upper branches had been pruned at least once were recognized as pollards. They were identified by all the main branches sprouting from single part of the trunk, mostly its swollen top. Trees bearing no signs of management were considered unmanaged (Figure 1). For each tree the diameter at breast height (DBH; 1.3 m above ground) and the presence/absence of hollows were recorded. Any cavity with an entrance hole larger than 5×5 cm and with the inner space larger than the entrance hole was recognized as a hollow. Where hollows were not accessible by ladder (>4 m above ground level), binoculars were used to search for hollows in trunks and branches (following Koch 2008 [28]).

To assess the effects of pollarding on tree growth, pollards and unmanaged trees were cored and their growth rates compared. Cores were taken from 10 unmanaged and 10 pollard trees using a steel increment borer (Mora™, Sweden) at the Pastvisko site, where one part of the formerly unmanaged willow stand was pollarded in 2003 (once only). All cores were dried, glued onto a wooden lath, cut off by razor blade and inspected for injuries, reaction wood and other aberrant features. One core was badly broken and another one contained rotten sections that did not allow successful ring width measurements. These two cores were removed from further analysis. For the remaining cores (n = 8 for pollard and n = 10 for unmanaged willows), rings were counted from pith to bark and their widths measured to the nearest 0.01 mm using the TimeTable measuring device and PAST4 software [29]. Ring sequences were cross-dated visually using the pattern of wide and narrow rings, and verified using the PAST4 program.

All necessary permits were obtained for the described field studies; the research was conducted under the permit No. 00356/KK/2008/AOPK issued by the Nature Conservation Agency of the Czech Republic. The data are deposited in Dryad, a publicly accessible digital repository [30].

### Data analysis

Relationships between DBH, the probability of hollow presence, and the effect of management (pollard/unmanaged) were tested. The data were analyzed using generalized linear regression models with binomial distribution (*logit* link) where presence/absence of hollow in the tree was a dependent variable, DBH (ln-transformed) was an explanatory variable (*lnDBH*), and management type (pollard/unmanaged) was a factor variable (*management*). An interaction between DBH and management type (*lnDBH:management*) was added to the model to assess the differences in slope shape between pollards and unmanaged trees. The second regression model where the interaction parameter was excluded was also tested, and then compared with the full model.

The effects of pollarding on tree growth rate were investigated by detection of radial growth in pollarded and unmanaged trees. Changes in incremental ring width were compared using a regression model with a repeated measures design. Each core sample (8 pollard, 10 unmanaged) was considered as a single subject, its ring widths being longitudinal measurements. In the model, ring width (*ringwidth*) was a dependent variable, subsequent years were equally spaced time points (*time*, continuous explanatory variable), and management type (pollard/unmanaged) was a factor variable (*management*). Firstly, the pattern in ring width increments for the period from 1990 to 2011 was tested, because from 1990 growth information for all the core samples was available. Secondly, the pattern for the period from 2003 to 2011 was tested, 2003 being the year in which trees were pollarded. In both cases, the interaction between *time* and management type (*time*:*management*) was included in the model. The quadratic effect of *time* was added to the model, and subsequently removed if not significant. The analyses were performed using R 2.14.2 software [31].

## Results

**Figure 2:**
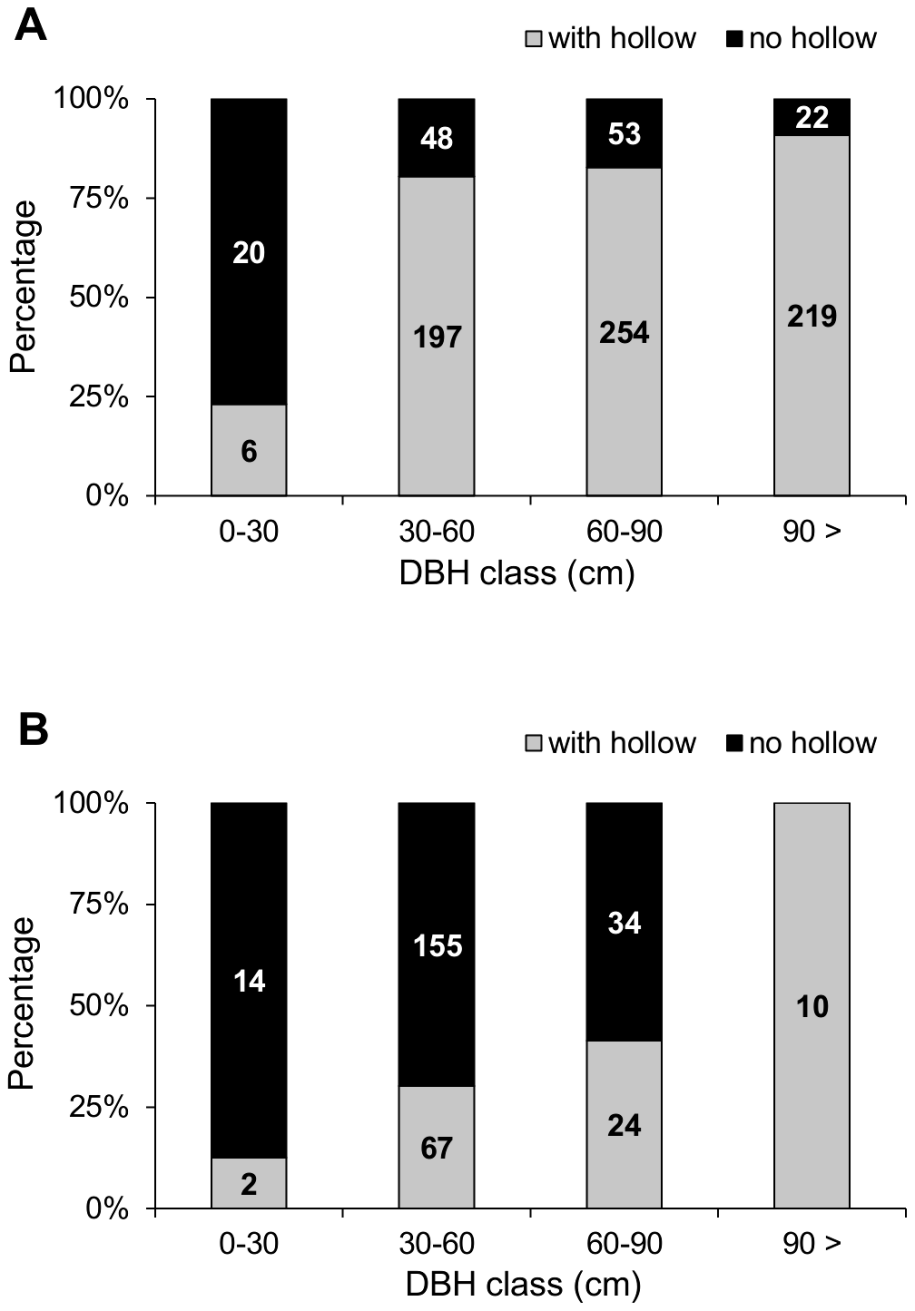
Proportions of hollowed trees in DBH classes. The proportion of hollowed willows in diameter classes (DBH class) for (A) pollards, and (B) unmanaged trees in the Dyje river catchment, Czech Republic. Numbers in bars indicate number of trees measured.

Data on 1126 willow trees were collected. Hollows were present in 677 (83%) out of 820 pollard trees, and in 103 (34%) out of 306 unmanaged trees. The number of trees examined and their mean DBH at each site are detailed in Table 1. The proportions of trees with hollows in each DBH class and for each type of management are shown in Figure 2. Six out of eight core samples obtained from pollards (nine years after single pruning), and one out of ten core samples obtained from unmanaged trees of the same age contained signs of wood decomposition (75% and 10%, respectively).

**Table 1:** Collected data on willows.

Site	Management	Number of trees examined	Percentage of trees with hollow	Mean DBH*		
				All trees	Trees with hollow	Trees with no hollow
Krive jezero	pollard	328	71	99	110	74
Vojkovice	pollard	492	90	64	66	43
Kanci obora	unmanaged	184	40	57	65	52
Pastvisko	unmanaged	122	24	46	49	45
Total		1126				

* Mean DBH  =  mean diameter in 1.3 m above ground.

Characteristics of pollards and unmanaged willows at four study sites in the Dyje river catchment, Czech Republic.

The hollow incidence increased with increasing DBH, and was affected by management as the regression models show (Table 2). Compared to unmanaged trees, the probability of hollow occurrence was higher in pollard trees, and especially in young trees, the increase in probability was steeper when backtransforming DBH to the original scale. The full model explained 23.1% of variance in the data, all three variables had a significant effect (*lnDBH*: χ^2^ = 172.71, d.f. = 1, P<0.001; *management*: χ^2^ = 145.34, d.f. = 1, P<0.001; *lnDBH:management*: χ^2^ = 4.21, d.f. = 1, P = 0.04). The simplified model without the interaction between *lnDBH* and *management* accounted for 22.9% of the total variance.

**Table 2:** Results of hollow presence analysis.

Coefficient	Estimate	SE	P
*A. Full model*			
pollard	−4.65	0.90	<0.001
lnDBH	1.48	0.22	<0.001
unmanaged	−6.29	2.23	0.005
lnDBH:unmanaged	1.10	0.55	0.047
*B. Interaction excluded*			
pollard	−5.44	0.83	<0.001
lnDBH	1.67	0.20	<0.001
unmanaged	−1.87	0.16	<0.001

Pollarding and DBH affect hollow occurrence in willows. Output of generalized linear regression models with binomial distribution (coefficient estimates are on *logit* scale), *lnDBH*  =  ln-transformed DBH, factor variable *management* is represented by its levels, 'pollard' and 'unmanaged'. Coefficients are displayed for (A) full model with interaction between variables (*lnDBH*: χ^2^ = 172.71, d.f. = 1, P<0.001; *management*: χ^2^ = 145.34, d.f. = 1, P<0.001; *lnDBH:management*: χ^2^ = 4.21, d.f. = 1, P = 0.04) and for (B) restricted model without interaction. For both models n = 1126.

**Figure 3:**
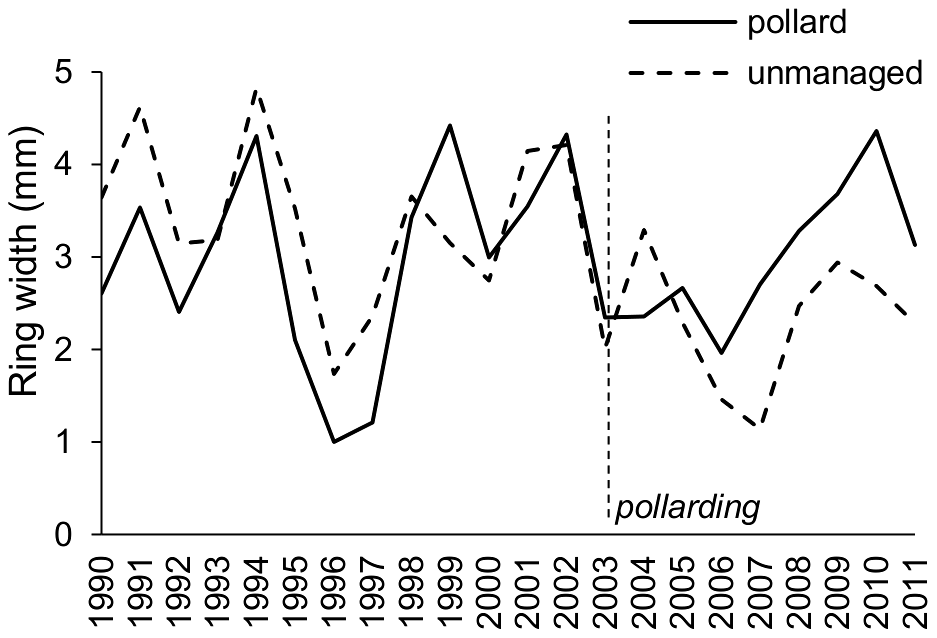
Radial growth of willows. Mean radial growth of pollards (solid line; n = 8) and unmanaged willows (dashed line; n = 10) at the Pastvisko site, Czech Republic. Although pollard willows show increased growth rates after the pollarding event of 2003 (vertical dashed line), the pattern was not significant in our study.

Growth rate did not differ between pollarded and unmanaged trees, as the results of the regression model for radial growth showed no significant difference in the incremental growth pattern. All effects proved to be non-significant in both the 1990-2011 model (*time*: F_1,392_ = 0.01, P = 0.26; *management*: F_1,392_ = 1.27, P = 0.98; *time:management*: F_1,392_ = 3.31, P = 0.07), and in the 2003–2011 model (*time*: F_1,158_ = 1.57,P = 0.21; *management*: F_1,158_ = 2.19, P = 0.14; *time:management*: F_1,158_ = 0.91, P = 0.34). Quadratic effects of time were not significant. The mean radial growth of pollarded and unmanaged willows in the period 1990–2011 is displayed in Figure 3.

**Figure 4:**
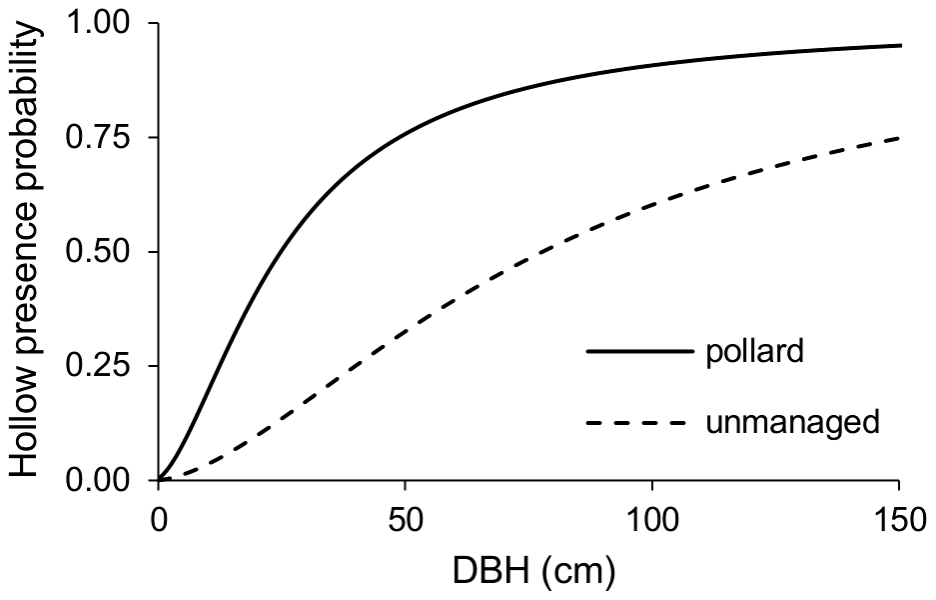
Predicted probability of hollow presence. Model predictions of hollow presence – probability that the tree with any given diameter (DBH) will contain a hollow, for pollards (solid line) and unmanaged willows (dashed line); (the *x-*axis is backtransformed to the original scale).

The probability of hollow occurrence in relation to DBH was predicted (Figure 4) using the simplified two-parameter model, because the significance value of the interaction parameter was low in the full model. The prediction showed that hollow occurrence was greater in pollard trees than in unmanaged ones.

## Discussion

### Study outcome and limitations

Our results show that pollarding increases the probability of hollow formation, especially in young trees (i.e., trees with small DBH). In pollard trees, hollows form more frequently and sooner than in unmanaged trees. For instance, in a stand of 100 willows with DBH of 50 cm, there would be ∼75 hollow trees if the trees were pollarded, and only ∼30 hollow trees if the trees were unpollarded (see Figure 4). Since pollarding speeds up hollow formation, it substantially increases the hollow density required for survival of hollow specialists [32].

Pollarding also has other positive effects. Firstly, the hollows in pollards occur in stems rather than in branches, while the latter prevail in unmanaged trees [33]. Stem hollows are larger, contain a greater volume of wood mould, and are thus able to host greater numbers of hollow specialists. Secondly, the bare, mostly sunlit heartwood after pruning is open to colonization by xylophages, including numerous threatened and protected species [23]. Pollards thus provide various deadwood microhabitats from a relatively young age. For example, at one of the sites (Vojkovice), larvae of *Osmoderma eremita* were found in a pollard <20 years old [10].

Owing to their lower height, pollard trees are less susceptible to branches breaking and falling due to weight imbalance, as long as pruning is carried out periodically. Pollards thus tend to live longer than unmanaged trees, and a single pollard may provide continuum of various deadwood microhabitats for hundreds of years [25]. On the other hand, if pollarding was discontinued, the increasing weight of overgrown branches often results in serious damage - trunk disintegration and the destruction of the tree. This is the reason why abandoned pollard stands suffer from rapid loss of hollow trees and subsequent loss of associated biodiversity [10].

It's not surprising that hollows are mostly present in trees with larger diameters [18], [34], [35]. As the tree ages, the heartwood becomes more susceptible to fungal infections and rot due to branch fall or bark loss [36]. Ranius *et al.* (2009) [18] provided data on hollow formation in oaks with regard to DBH. Of oaks with a diameter of 80–100 cm, 20–50% contained hollows. In our study, hollow incidence in both unmanaged and pollard willows of the same diameter is much higher than in oaks, probably due to the fact that faster growth and softer wood make willows more prone to damage and infections [4], [37].

During the last century, pollarding was abandoned in most of the study area and also in most of Europe [11], [24], [25], [27], [38]. Because of this, it is difficult to separate the effects of pollarding and tree age. Generally, old trees have been pollarded in the past, whereas young trees never have. In the study area, pollarding has continued locally, but is concentrated on predominantly older trees. As a result of this, our sampling was limited by the absence of stands containing both unmanaged and pollard willows of a similar age. This might possibly be a source of some unintended bias and further studies would therefore be needed to ensure sufficient separation of the age effect.

We found no effect of pollarding on radial tree growth. This is in contrast with the documented decrease in growth rate after pollarding in oaks [39]. Willows, however, may respond to pollarding in a different way than oaks. Also, the sampled trees grew in dense stands, and were probably affected by competition. In easily resprouting, light-demanding willows, reduced competition after pruning (pollarding) may have balanced the negative impact of branch removal. Larger sample size would be needed in order to test such a hypothesis. The poor quality or absence of central heartwood in regularly pollarded older trees also prevented us from assessing the potential effects of regular pollarding on tree growth. This question has yet to be addressed.

### Traditional management as a part of landscape history

Ancient landscape in Europe was strongly influenced by human activities over long periods of time [4]. In the past, traditional pruning techniques like pollarding, shredding or coppicing were used for firewood and fodder production [40]–[42], and forests were also used for livestock grazing [43], [44]. As a result, old, open-grown and hollow trees were common features of the landscape.

Today, the biota associated with old and/or hollow trees finds refuge mainly in orchards, alleys, parks, pasture woodlands and game reserves rather than in intensively managed forest and agricultural landscape, or strict forest reserves [26], [45]. The importance of such man-made habitats is especially emphasized for the noble chafer beetle *Gnorimus nobilis* [46], [47] and for *Osmoderma eremita* [48]. The latter is among the best known European beetles and serves as an indicator and umbrella species for hollow-dependent invertebrates [49]. Both beetles are thermophilic and rarely occur in natural or semi-natural forests that are too shaded [47], [48]. Hollow-dependent invertebrates often have low dispersal abilities, and thus require a high density of microhabitats in space and time [12], [13]. Traditional pollard stands, pasture woodlands, and orchards with large aggregations of sun-exposed hollow trees provide suitable conditions.

Other studies provide evidence for the importance of traditional management practices as they stimulate the formation of open forest features [50]. The cessation of pollarding was found to have negative effects on assemblages of lichens and epiphytic vegetation [25], [38], [51]. Robles *et al.* (2011) [52] found that the biodiversity of secondary cavity nesting birds was greater in oak forests traditionally managed by extensive grazing and cutting for firewood than in dense forests. Following the same principle as pollarding, coppicing most likely increases the incidence of hollows near the ground, and its cessation thus threatens the violet click beetle *Limoniscus violaceus*, a rare and highly endangered species, which develops in the wood mould of basal cavities. The beetle can mostly be found in formerly coppiced forests [53], [54] or ancient forest pastures [55].

In Europe, traditional silvicultural practices have contributed to the creation of today's rare deadwood microhabitats, and therefore they helped to preserve saproxylic species over the years. The discontinuation of such practices not only threatens numerous saproxylic organisms in commercial woodland, but it also leads to a peculiar situation in many protected areas: old trees, that currently host populations of hollow-dependent and other specialised saproxylics, were often subject to pollarding or coppicing, that increased dead wood availability. Young trees, on the other hand, were not treated in this way. This is likely to result in the reduction of suitable deadwood microhabitats in the near future.

Conservation practitioners, therefore, need to acknowledge that extant populations of endangered saproxylic species, many of them preferring sunny conditions, very likely depend on microhabitats created by former management practices, since abandoned [5]. Although the insects are still present at numerous sites, they survive only temporarily, as a part of extinction debt created by past management changes [56], [57]. The importance of active management practices is, however, rarely appreciated. Nature conservationists often praise natural, semi-natural and 'old-growth' habitats, and tend to ignore site history and the habitat requirements of endangered organisms. In particular, the 'strict forest reserve' concept [58], [59] based on non-intervention management in woodland habitats is especially damaging when applied to traditionally managed sites [3], [43], [60]–[62]. In Europe, natural disturbance factors such as large herbivores [63] and fires [64] are mostly lacking. Natural succession thus leads to greater canopy closure and causes a transformation from low competition and sunny conditions to higher competition with shady and cold conditions. Old and veteran trees are not able to adapt to the new conditions and subsequently die [4], [5], which leads to the loss of important deadwood microhabitats and therefore to a decline in saproxylic biodiversity associated with these trees.

### Future perspectives

The promotion of evidence-based conservation management targeted at saproxylic species is one of the major topics of the currently emerging European Saproxylic Beetle Conservation Strategy [3]. Conservation and retention of hollowed trees as one of the specific habitats are therefore of crucial importance. Creating artificial habitats might be necessary, especially when it takes a long time for the natural production of such habitats [19], or if factors contributing to their formation in the past, e.g. grazing by large herbivores or fires [63], [64], are missing. Active management, therefore, should be adopted to prevent the gradual decimation of saproxylic biodiversity. As shown in the present study, pollarding might play an important role in the restoration of saproxylic habitats. If trees along watercourses, roads and other linear structures in the landscape were to be pollarded, they could well become important habitats and corridors connecting refugia of saproxylic fauna.
